# Multimodal dataset of real-time 2D and static 3D MRI of healthy French speakers

**DOI:** 10.1038/s41597-021-01041-3

**Published:** 2021-10-01

**Authors:** Karyna Isaieva, Yves Laprie, Justine Leclère, Ioannis K. Douros, Jacques Felblinger, Pierre-André Vuissoz

**Affiliations:** 1 Université de Lorraine, INSERM, IADI, Nancy, F-54000 France; 2 Université de Lorraine, CNRS, Inria, LORIA, Nancy, F-54000 France; 3 Oral Medicine Department, University Hospital of Reims, 45 rue Cognacq-Jay, 51092 Reims, Cedex France; 4 CIC-IT, INSERM, CHRU de Nancy, Nancy, F-54000 France

**Keywords:** Communication, Oral anatomy

## Abstract

The study of articulatory gestures has a wide spectrum of applications, notably in speech production and recognition. Sets of phonemes, as well as their articulation, are language-specific; however, existing MRI databases mostly include English speakers. In our present work, we introduce a dataset acquired with MRI from 10 healthy native French speakers. A corpus consisting of synthetic sentences was used to ensure a good coverage of the French phonetic context. A real-time MRI technology with temporal resolution of 20 ms was used to acquire vocal tract images of the participants speaking. The sound was recorded simultaneously with MRI, denoised and temporally aligned with the images. The speech was transcribed to obtain phoneme-wise segmentation of sound. We also acquired static 3D MR images for a wide list of French phonemes. In addition, we include annotations of spontaneous swallowing.

## Abstract (summary)

**Table:** 

Measurement(s)	Vocal tract images • Speech
Technology Type(s)	Magnetic Resonance Imaging • Microphone Device
Sample Characteristic - Organism	Homo sapiens

Machine-accessible metadata file describing the reported data: 10.6084/m9.figshare.16404453

## Background & Summary

The investigation of the movement of speech articulators has a number of applications including study of speech production^1^, speech recognition^2^, as well as some medical applications: diagnosis and rehabilitation of abnormal speech and swallowing, study of orto-facial structures implicated in sleep apnoea syndrome^3^. Information on motion can be obtained using different methods including electromagnetic articulography (EMA)^4^, X-ray^5^ and ultrasound imaging^6^. Nowadays, magnetic resonance imaging (MRI) holds one of the leading positions as a data acquisition method in speech sciences^7–10^ due to its non-invasiveness and absence of long-term health hazards. Contrarily to other techniques such as ultrasound, which fails to visualise the articulators separated from the sensor by air, or EMA which provides only the sensors’ trajectories glued on the upper vocal tract articulators, MRI succeeds to visualise the whole vocal tract.

However, MR imaging of a speaking person is a challenging problem due to the fast motion of articulators. One of the techniques allowing a reasonable spatio-temporal resolution of recorded speech, is cine-MRI^11,12^. However, this method requires several identical repetitions of the same target utterance, which leads to artifacts in case of non-periodicity, and increases acquisition time. Real-time MRI allows high spatio-temporal resolution without repeating and is usually based on spoiled gradient echo sequences^8,13,14^. Acquisition can be sped-up by usage of non-cartesian (generally undersampled) schemes which ensure good coverage of the k-space centre. This approach has been employed by several research groups to study speech. A spiral encoding scheme was applied in^15,16^. and was thereafter combined with sparse-SENSE constrained reconstruction methods^8,17^. In^18,19^, a radial encoding scheme was used together with a compressed SENSE reconstruction. The technique^20^ makes use of radial sampling and the regularized nonlinear inversion reconstruction. Another approach, which does not necessarily assume a non-cartesian encoding, was used for dynamic 3D imaging of the vocal tract^14,21^.

Since the technologies listed above are not easily available, data sharing could greatly accelerate research in the field. In this context, multiple databases exist for English speakers. Real-time MRI datasets, where 460 sentences were pronounced by 4 and 10 speakers, are presented in^22^ and^16^, respectively. The databases^23^ and^24^ which were acquired from 17 and 8 speakers respectively, include both real-time and 3D static MRI. An emotional speech dataset recorded from 10 speakers was published in^15^. Recently, an extremely rich dataset counting 75 English speakers was presented^25^. However, MRI datasets representing other languages are very limited. 2D dynamic MRI with temporal resolution of 7 frames per second of one female Portuguese speaker was published in^26^. Static 3D MR images of five Japanese vowels pronounced by one male speaker are presented in^27^. A dataset of 3D vocal tract shapes was published recently^28^ for two German native speakers. A 2D dynamic with 3D static MRI database including 2 male French speakers was also acquired earlier^29^. Nevertheless, the available data does not allow exhaustive investigation of these languages. Moreover, all the existing publicly available databases offering high spatio-temporal resolution dynamic MRI, exploit similar acquisition technologies due to the fact that they are acquired by the same research team. Availability of datasets of different qualities could serve to get better precision in some aspects.

In this work we report on a multi-modal MRI database consisting of 2D real-time and 3D static MR images of the vocal tract of 10 French speakers. The protocol used for the real-time MRI acquisitions for our dataset was successfully used by multiple groups in the context of the study of the articulators’ motion^30–33^. While performing investigations on the vocal tract organs, it is crucial to consider the diversity of their movements during speech production. Standard French language includes 35 phonemes (18 consonants, 14 vowels, 3 semi-vowels) which form 1290 diphones^34^ and many complex consonant clusters. To cover this variability as much as possible, a corpus was previously developed^29^. The corpus allows to explore numerous phenomena specific to the French language such as nasal vowels, uvular /ʁ/^35^, French /y/, short /ɥ/, and strong anticipation of labial features^36^. The dataset includes annotations of the speech and of spontaneous swallowing and will thus provide researchers with data having a good coverage of the French phonetics to further explore French speech production and physiological processes taking place in the vocal tract vicinity.

## Methods

### Participants and speech task

**Fig. 1:**
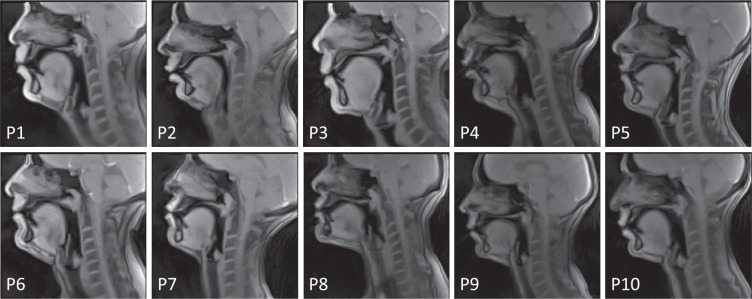
Examples of real-time images of all ten speakers pronouncing /u/ (“filou’ from the first sentence).

The participants were 5 male and 5 female native French speakers (aged 29 ± 8 years) without any speech or hearing problems. Presence of any metal in the vocal tract vicinity, which may generate susceptibility artifacts, was also an exclusion criterion. Relevant patient characteristics are listed in Table 1. A set of mid-sagittal images demonstrating speakers’ anatomy is presented in Fig. 1.

**Table 1:** Relevant speakers’ information.

Patient Code	Gender	Age	Height, cm	Weight, kg
P1	Male	25	175	70
P2	Male	22	180	73
P3	Male	41	178	68
P4	Female	45	174	78
P5	Male	20	175	70
P6	Male	27	172	73
P7	Female	24	178	59
P8	Female	25	168	68
P9	Female	33	162	52
P10	Female	30	170	53

All participants provided written informed consent, including written permission to publish the materials of this experiment. The data was recorded under the approved ethical protocol “METHODO” (ClinicalTrials.gov Identifier: NCT02887053). The study was approved by the institutional ethics review board (CPP EST-III, 08.10.01).

The previously designed corpus^29^ was presented in form of a pdf-file (see Supplemental Materials) which was projected on a screen in the MRI room so that a speaker could read it during the experiment without difficulty. The corpus included two parts. The first one served for the acquisition of dynamic 2D data and included 77 sentences which were constructed to provide an almost-exhaustive coverage of the French phonetic contexts of vowels /i,a,u,y/ and some nasal vowels selected from /$\widetilde{\alpha },\;\widetilde{{\rm{o}}},\;\widetilde{{\rm{\varepsilon }}}$/. Several levels of criteria were used to guide the manual construction of those sentences. After the insertion of a new sentence the first level of criteria evaluated was the number of VV for all the vowels, the number of CV for C in /p, t, k, f, s, ∫, l, ʁ, m, n/ and V in /i, a, u/ plus /y/, the number of VC with C as a coda and C in /l, ʁ, n, m/ and V in /i, a, u, y, e, ɛ, o, ɔ/, the consonant clusters C1C2V with C in /p, t, k, b, d, g, f/, C2 in /ʁ, l/ and V in /a, i, u, y/ (the other CCV following the same pattern with /s, ∫, v/ are rare in French), and VC, of C in a coda, and 15 complex consonant clusters (at least a sequence of 3 consonants, between two vowels). Except for those clusters and with very few exceptions all the contexts appear within words to avoid the effect of prosodic boundaries. This first level of criteria covers the very heart of the corpus in terms of mandatory phonetic contexts. We wanted well-constructed French sentences and therefore words not corresponding to the target contexts were added. They provide new contexts, and in particular contexts with vowels outside the set of cardinal vowels plus /y/. VCV are counted by considering groupings of close vowels. There are 6 groups of vowels (/i, e/, /ɛ, a/, /u, o, ɔ/, /y, ø/, /œ, ə/ and nasal vowels /$\widetilde{\alpha },\;\widetilde{{\rm{o}}},\;\widetilde{{\rm{\varepsilon }}}$/. This provides a second level of evaluation which helps required words to build well-constructed sentences. Other words required to form well-constructed sentences provide additional contexts with the remaining vowels. All the words (except “cartoons” and “squaw”) are of French origin to avoid any ambiguity of pronunciation. The sentences were divided on groups of 3–5 to ensure comfortable duration of a session. The speech task for 2D real-time acquisition is presented in Online-only Table 1.

The second part comprised 3D static data acquisition and included 5 silent acquisitions with different positions of the tongue (against the upper teeth, against the lower teeth between the incisors, retroflex and deep retroflex), 14 vowels and 12 consonants (each in context of several vowels). The full set of the phonemes and positions included into the speech task for 3D static acquisitions, is listed in Online-only Tables 2 and 3. The first three positions were designed to help the registration of teeth within MRI data. The participants were asked to keep the same position before and up to the end of the MRI noise in case of silent positions or vowels. For a consonant C in context of a vowel V they were instructed to phonate V until the end of the countdown of an MRI operator who then started a sequence, then to keep the articulation of C until the end of the MRI noise and then phonate V again (the consonant production in this case is called blocked articulation). This helped speakers reach the expected articulatory position for each of the consonants articulated within a given vowel context. Duration of each sequence for the static 3D data acquisition was chosen to be 7 seconds, as a compromise between the volunteers’ comfort and the image quality. While sometimes it was not very natural to keep the same position for such time, especially in the case of plosive consonants, the task appeared to be absolutely feasible.

The presentation was sent to the volunteers before the experiment so that they had at least one day to get familiar with the sentences which sometimes sound strange even if they are all well-formed French sentences. Additionally, the speakers were carefully instructed directly before the acquisition to guarantee correct understanding of the task.

### Data acquisition and alignment

The MRI data was recorded at Nancy Central Regional University Hospital on a Siemens Prisma 3 T scanner (Siemens, Erlangen, Germany). The speakers were in supine position and the Siemens Head/Neck 64 coil was used.

For the 2D real-time we used radial RF-spoiled FLASH sequence^13^ with TR = 2.22 ms, TE = 1.47 ms, FOV = 22.0 × 22.0 cm, flip angle = 5°, and slice thickness was 8 mm. Pixel bandwidth was 1670 Hz/pixel. Image size was 136 × 136, and in-plane resolution was 1.6 mm. Images were recorded at a frame rate of 50 frames per second and reconstructed with a nonlinear inverse technique presented in^13^. This method represents a formulation of a nonlinear optimisation problem with respect to both image and coil sensitivity maps which is solved iteratively with regularized Gauss–Newton method. The protocol used for dynamic data acquisition differs from the protocols of the published publicly available databases, and thus the quality is also somewhat different. Radial encoding trajectories are shorter than spiral ones, so that the repetition time is lower in our case (2.22 ms comparing to 6.004 ms in the latest dataset^25^) which probably decreases the manifestation of the off-resonance effects. Also, some residual aliasing artifacts can be remarked in case of the datasets^16^ and^15^. However, due to the larger slice thickness (8 mm comparing to 6 mm), the partial volume effects are more pronounced in our case. Protocol^17^ proposes higher temporal resolution (83 frames per second^24,25^), while the protocol used in our case offers higher in-plane spatial resolution.

**Fig. 2:**
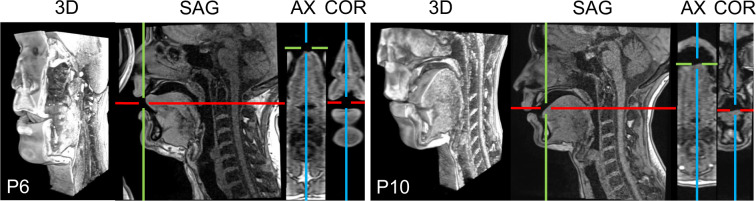
Examples of 3D static images: P6 pronouncing /l/ in context of /a/, and P10 pronouncing /ã/. The 3D volumes were cropped for better visibility.

For 3D static data, we used 3D VIBE with TR = 3.8 ms, TE = 1.55 ms, FOV = 22.0 × 20.0 cm^2^, flip angle = 9°, slice thickness was 1.2 mm, and in-plane resolution was 0.69 × 0.76 mm^2^. Pixel bandwidth was 445 Hz/pixel. Image size was 320 × 290 with 36 slices. Acceleration factor was iPAT = 3. Each sequence had duration of 7 seconds. Examples of the resulting 3D images are given in Fig. 2.

Audio was recorded at a sampling frequency of 16 kHz inside the MRI scanner by using a FOMRI III optoacoustics fibre-optic microphone (FOMRI III, Optoacoustics Ltd., Mazor, Israel) placed in the scanner. The volunteers wore earplugs to be protected from the scanner noise, but were still able to communicate orally with the experimenters via an in-scanner intercom system. Since the sound was recorded at the same time with the MRI acquisition, some additional noise is present in the audio signal. In order to suppress the noise, we used the algorithm proposed in^37^. The algorithm relies a on the hypothesis that a noisy sound represents a gaussian mixture of two components (voice and MRI noise in this case), and the decomposition is based on an expectation-maximisation algorithm. The components are characterized by some source and resonator spectral features. A clear speech recording, which was required by the algorithm, was done separately for each speaker just before the dynamic MRI acquisition starts, with the same patient and microphone positions.

**Fig. 3:**
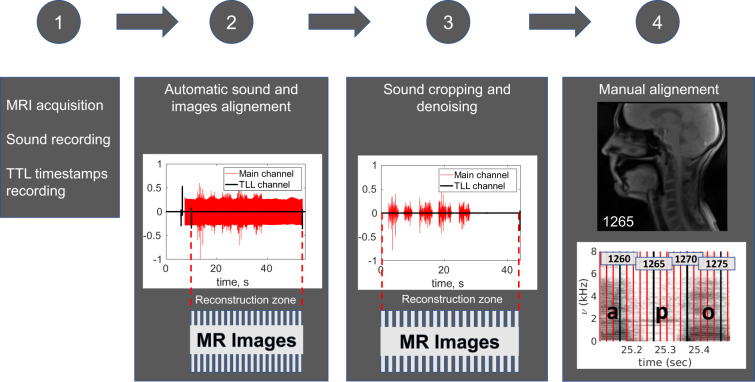
Data processing routine illustrated with the data of P9. The figure should be read from left to right, and each item corresponds to a processing stage. The first item lists the acquired data, second and third items illustrate sound alignment and denoising results, and the last item aims to explain the manual alignment principles. The articulators’ position on the MR image 1265 corresponds to the onset of /p/ (the lips have just come in contact). The plot below the MR image shows a spectrogram of the sound with vertical lines denoting centres of MRI acquisition intervals (each 5-th line is black for better readability). From the spectrogram, the image 1265 is supposed to correspond to the middle of /p/, which is not the case. The sound is haste by approximately 3 images = 60 ms and thus the sound and the images should be re-aligned.

In order to align the sound with MRI data, we used Signal Analyzer and Event Control system (SAEC) which had previously been designed^38^. We applied it to record timestamps of the reconstruction start and the sequence stop events and send them to a channel of the opto-acoustic system which allows MRI transistor-transistor logic (TTL) commands recording. This enabled automatic synchronisation of the images and the sound. However, during manual examination it was found that the images are somewhat shifted with respect to the sound after the automatic alignment. A shift of 2 images (40 ms) was explained by the application of the temporal median filter of width 5 (100 ms) during reconstruction. Nevertheless, due to some temporal variations of the TTL signals and/or sound reception, probably caused by USB jitter, the temporal shift between the images and the sound slightly varied from series to series. Thereby, the shifts were required to be manually defined for each series by comparison of sound amplitude and/or spectrograms with MR images. From a practical point of view, for this purpose we selected the events which are clearly visible on both the acoustic signal and the images: onsets of the sounds /p/ and /b/. This event corresponds to the time moment where the lips contact occurs and results in an abrupt and considerable acoustic signal weakening. In most of the recorded sequences there were /p/ and /b/ near the recording extremities. If this was not the case, the fallback solution was to use the contact between the tongue tip and teeth alveoli for /t/ and /d/ which also corresponds to a strong weakening of the signal. Thus, the full pipeline consisted of four steps. 1. Data acquisition. 2. Automatic alignment using the TTL timestamps. 3. Sound cropping, so that it fits the MRI acquisition time interval, which was necessary for the denoising, and the denoising itself. 4. Manual shift determination and re-alignment. The illustration of the different steps of the alignment routine is given in Fig. 3.

### Transcription of the continuous speech corpus

Speech transcription is the temporal sentence-wise, word-wise or phoneme-wise segmentation of an audio recording. Together with the synchronisation, it provides correspondence between images and pronounced phonemes which is helpful for many practical applications. Even though the speech task was pre-defined, some speakers made mistakes or repeated some syllables or words. To take these deviations with respect to the expected pronunciation into account, each recording was inspected by an investigator and all the hesitations or repetitions were included to the text used for the alignment. The transcription was done in two steps. First, the sentences start and end were manually annotated from the sound, which was denoised and cut to fit the MRI acquisition time interval (see Fig. 3). This was done using Transcriber 1.5.2 (http://trans.sourceforge.net/en/presentation.php). This software generates .trs files which include the timestamps and the corresponding text. Text annotations and audio signal were synchronized by a forced alignment automatic speech recognition system Astali (http://ortolang108.inist.fr/astali/) trained on French. It used the .trs text annotations together with the denoised sound to perform the temporal segmentation (both word-wise and phoneme-wise). The phonemes are stored inside a file using SAMPA phonetic annotation system^39^.

### Swallowing detection

Swallowing is defined as a series of mechanisms allowing transportation of food, drinks or saliva to the stomach. This mechanism occurs in four steps: (1) A pre-emptive phase, with lip closure; (2) an oral phase, which corresponds to the bolus transportation from the front to the posterior area of the oral cavity, in order to reach the pharynx; (3) a pharyngeal phase, where the bolus continues its way through the pharynx, and (4) an oesophageal phase allowing the bolus to penetrate into the stomach.

The oral phase is particularly interesting, owing to the complexity of the muscle contractions and anatomical movements achieved during this step. During this phase, which lasts about one second, the mandible is stabilized when the teeth are in contact and in maximal intercuspation occlusion (MIO), after lip closure. At the same time, the tongue initiates a propulsion movement which begins in the anterior hard palate, and then performs a second contraction to propel the bolus at the rear up to the pharyngeal areas. Following this contraction, the oropharynx is closed to prevent bolus penetration into the upper airways (UA). The bolus can then continue its way to the pharynx.

Swallowing pathologies are numerous, and imaging devices remain limited to study in real-time physiological movements of the anatomical structures of the upper airways. In order to facilitate this observation and identify the oral phase of swallowing, we propose a protocol to determine the start and end positions of swallowing on our MRI images in real time. (1) The image counting starts when the apex of the tongue touches the hard palate. However, for some images recorded during speech, the tongue might already be in this position while swallowing begins. In these cases, we took as a landmark the most anterior contact of the dorsal part of the tongue with the hard palate. (2) At the beginning of the oral phase of swallowing, the elevation of the hyoid bone is observed. The end of the oral phase has been described as the moment when the space between the tongue and the velum and/or the soft palate reappears. At the same time, the hyoid bone returns to its initial position, and the oropharynx relaxes to allow the ventilatory flow to recover.

## Data Records

The data is available on figshare^40^. Each of the 10 folders contains the data of a speaker. To summarize, a folder (except the second one) contains 16 dynamic and 76 static series. Each of the dynamic series counts 1800 to 2200 frames and has duration about 1 minute, which results to overall 34800 images (approximately 15 minutes) per subject. Each static series consists of 36 slices.

The dataset is organized as follows: the root directory contains ten speakers’ folders with names “PXX” (XX here is a patient code). The speaker data is divided into three folders: DCM_2D, DCM_3D and OTHER. Inside DCM_2D and OTHER folders there are 16 subfolders with names SYY (YY is a series number) which correspond to the different dynamic acquisitions. Files stored inside the subfolders of DCM_2D are DICOM files with the MRI 2D dynamic data, and files contained inside subfolders of the OTHER folder are the corresponding denoised cropped sound, TEXT_ALIGNMENT_PXX_SYY.trs file (sentence alignment), TEXT_ALIGNMENT_PXX_SYY.textgrid file (alignment of words and phonemes), SWALLOWING_PXX_SYY.trs which has the same format as “sentence alignment” and contains swallowing timestamps, and an example video VIDEO_PXX_SYY.avi generated with the provided code (after compression).

The 3D static data can be found inside the subfolders of the DCM_3D folder. The name of those subfolders corresponds to the target phoneme and possibly its vowel context, for instance “tu” for /t/ in the vocalic context of /u/. There are also three static positions to help determine the position of teeth which are not visible on MRI scans. They are denoted as UP (for the tongue touching the upper incisors), DOWN (for the tongue touching lower incisors) and CONTACT (for incisors in contact). The latter was used to check. consistency of the teeth positions (as determined by UP and DOWN).

**Fig. 4:**
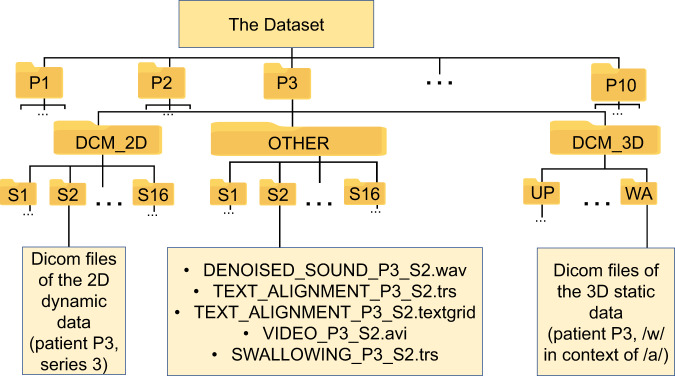
The dataset structure illustrated with the example of the second dynamic series and the 3D static image of the consonant /w/ in context of /a/ of the P3 data.

The correspondence between the folder names and the speech task performed in frames of an MRI series, is given in the Online-only Tables 1–3. The dataset structure is also illustrated in Fig. 4.

The text alignment data are the Transcriber sentence annotation files (.trs files) which include pronounced text and the timestamps of start and end of each sentence on the one hand, and word and phoneme segmentation files (.textgrid Pratt files)^41^ on the other hand. The code which allows reading these files is provided.

For P2, 3D static images of consonants phonation were not acquired because of technical reasons, thus only dynamic 2D data and 3D static images of vowels and silent positions were included. All other speakers performed the full list of acquisitions.

## Technical Validation

**Fig. 5:**
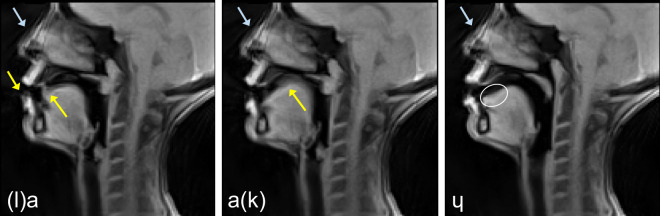
Artifacts and imperfections that can occur illustrated on the images of P7. Blue arrows point to an aliasing artifact, which, howeher, does not affect the quality of the vocal tract imaging. Yellow arrows point to motion artifacts that take place in case of rapid change of the articulators’ position (like the transition from /l/ to /a/, and from /a/ to /k/). The white ellipse points to a tongue region with slightly lower intensity which is caused by relatively large slice thickness and tongue shape variations in the left-right direction (partial volume effects).

The dynamic 2D images were visually inspected by the researchers. In general, the images quality was good. Some minor artifacts can be observed as it is pointed out in Fig. 5. Some partial volume effects occurred because of relatively large slice thickness (8 mm). Despite the temporal filter, some residual radial aliasing artifacts were observed. Very fast articulatory gesture can lead to blurring, for example when the tongue tip approaches the alveolar region for /t/, /d/ or /l/.

The video-sound alignment was verified by a researcher having more than 20 years of experience in the field (author Y.L.). This was done by cross checking of the sound track and images using the videos included to the database as described in Methods section. In the case a misalignment found, a proper shift was applied to the sound.

The text alignment was checked by authors K.I., P.-A.V. and Y.L by comparing the sound, the images and the text. Some errors caused by the fact that certain speakers made many pronunciation errors and/or hesitations were generally corrected, however some residual errors can still have place. The list of the corrected mistakes is presented in Online-only Table 4. An experienced reader can see that there are some minor phoneme-wise sound segmentation errors (order of 1 frame which corresponds to 20 ms) in the case of plosive consonants (explained by the fact that almost no sound is produced). We chose to keep the automatic annotations.

The vocal tract shape during 3D static data acquisition, which should correspond to a required phoneme, was visually inspected directly during the course of the experiment by authors K.I. and P.-A.V. In case of obviously wrong positions, the data was reacquired up to three times. The resulting 3D images were checked by author Y.L. The cases of phoneme/image inconsistency for the static 3D data are given in the Table 2. In addition to individual comments given in the Table 2, here are some general trends. Blocked articulations (freezing a position just before producing a consonant) is not a natural gesture in speech production. It is especially difficult to control the velum position since there is no acoustic feedback. This explains why the velum is in a lower position in some cases where it is expected to be in a higher position, for instance stop consonants. Some speakers who were not familiar with phonetics were unable to respect the instructions and despite several explanations, they did not understand how to do reach the expected articulatory positions, especially those corresponding to stop consonants. For the same reason, subjects reached a far better articulatory position for phonated items, (i.e. vowels and fricatives) simply because the condition is more natural. The strong MRI noise probably strengthened the Lombard effect for vowels which slightly changed the articulation. We decided to keep this inconsistent data, since it can still have some applications, i.e. as dataset augmentation in case of machine learning.

**Table 2:** Evaluation of articulatory shapes produced.

Subjects	Comments
P1	Good images. Very extreme retroflex shape.
P2	Only some images were recorded because of a technical problem.
P3	The subject did not understand instructions correctly (no contact for stops, lips not closed for /p/) but sustained sounds, i.e. vowels and fricatives, are correct.
P4	The overall quality of images is not very good. Oral vowel shapes are correct, and in to a lesser extent fricatives. Many blocked articulations do not exhibit expected features (contact for stops, velum position…).
P5	Very good images. /li/ was not articulated correctly.
P6	Good images. Slight move for /a/ and /mɔ̃/.
P7	The contact between the fixed and the mobile articulators is not reached for several stop consonants. The velum is in the upper position for /m/, under-anticipation of the tongue position for /p/, exaggeration of the tongue tip position between upper and lower teeth for /t/, no contact at the place of articulation of some /k/. The articulators’ positions are, in general, not very natural.
P8	Strong forward position of the mandible. Instead of being in the upper position the velum is in the lower position for many oral articulations. Several stop articulations without contact between the fixed and mobile articulators (for /p/ and /t/).
P9	The subject did not understand instructions despite several trials. Some vowels are articulated with the mouth closed and some tongue shapes are very unusual for /k/ which has not been articulated correctly.
P10	Good images. The velum is sometimes in the lower position (/a/ and /la/ for instance) for oral sounds.

## Supplementary information

Supplementary Material

## Data Availability

A MATLAB code which generates a video from the dynamic data is available on GitHub https://github.com/IADI-Nancy/ArtSpeech. This code also allows reading alignment annotation .trs and .textgrid files, and reading audio and dicom files. It uses mPraat third party toolbox which is available on bbTomas GitHub https://github.com/bbTomas/mPraat. The code was tested on Linux distribution of MATLAB2018b and MATLAB2020a. A Python toolbox for .textgrid files parsing also exists^42^.

## Online-only Tables

**Online-only Table 1:** Speech task for the 2D real-time MRI.

Folder name	Speech task
S1	1) Le filou et la fripouille manipulent de l’acrylique antirides dilué sous le tipi. 2) En haut du cumulus Pierre prit dix choux, du rouge et du clafoutis puis se camoufla en clown. 3) L’actionnaire des yaourts Caprice des Dieux couvrit le doigt sur le cahier aimanté. 4) Ne repoussez pas l'écrou de la galtouse de ris à la pomme.
S2	5) Du coup l’oculiste tout fou dévissa sans scrupule le volant du véhicule. 6) La ciguë de l’homme de loi roux est dans la grue sur le parking. 7) Il n’a pas voulu, ou n’a pas pu injecter un sous-multiple de la dose en sous-cutané. 8) Je veux annuler pour éviter le raffut du saut dans le grand bassin. 9) Plus nous y croyons pire est le trou dans le lit du pauvre.
S3	10) Trois sacs carrés. Trois sacs carrés. Trois sacs carrés. 11) Vous dactylographiez sa soupe sirupeuse au lit. 12) Le chouan qui parle wolof et a l’ouïe fine prépare une mixture bien pire. 13) Le chouchou du fou truqua le chargeur du fusil de leur nounou taciturne. 14) Sonne le glas à plat sans faire glouglou dans le foin et les plumes.
S4	15) Le stupide toutou sous-nutri anticipa couci-couça l’africanisation des bikinis. 16) Les attabler. Les attabler. Les attabler. 17) Il pouffa quand il ouvrit l’incunable qui montrait un prunus et les outils des Manouches. 18) Nous galopâmes avec peine jusqu’au bout sous le soleil. 19) Elle l’accuse de la diffamer en disant qu’elle a couru et s’est amusée avec du ciment et du sable humide.
S5	20) J’exultais car elle joue et fume comme jamais avec les poules. 21) Crabes bagarreurs. Crabes bagarreurs. Crabes bagarreurs. 22) Il a pourri. Il a pourri. Il a pourri. 23) Nous analysions avec courroux l’humus du bois touffu, où tu voyais des bombes antichars et des coucous. 24) En écoutant la fl û te, le chevreau mangea la robe à froufrous de Maurine.
S6	25) Lui as-tu pris ta presse pour les piles du roi des Zoulous? 26) Elle culbuta et accoucha huit fois dans les choux de Gilles. 27) Drapé dans son manteau mais pas du tout alourdi par le poids du chat il dut chuter sur la mosaïque. 28) Paul jugeait le Vésuve sans danger depuis le môle. 29) Il sut ça si tôt, qu’il fit tout pour diffuser les coupures à ras bord.
S7	30) Des nuages gris et un cyclone destructeur s’approchent du groupe polaire. 31) Je vois le loubard, le wagon et des ficelles qui chutent dans la rivière. 32) Il l’a daté. Il l’a daté. Il l’a daté. 33) A la cantine, un Druze cache ses frasques et ses vices, en fricotant avec un plouc. 34) Au bilan, les députés juxtaposeraient la sous-poutre.
S8	35) Pour tout casser. Pour tout casser. Pour tout casser. 36) Amoindri par les tirs, le flibustier vadrouille à hue et à dia sans détour. 37) Finalement le loup du roi a vu la squaw redoutée des alouettes de Laval. 38) Elle moulut du pou chilien et du loup pour les enfants affamés du ru. 39) L’azimut chimique partira sans hachurer les sinus acquis avec humour pendant la pénurie.
S9	40) L’aménageur qui est venu cherchait l’anthologie des appareils se réparant seuls. 41) L’ouvrage qui disposait d’une boussole était carbonisé de part en part. 42) Le premier des voyous ment très fort avant de souffler sur le nageur. 43) Il zappe pas mal. Il zappe pas mal. Il zappe pas mal. 44) Comme alternative, j’ai agglutiné des tours de fil pour avoir un aimant supranaturel.
S10	45) L’exclusivité fait peur à l’administrateur de biens du port. 46) Où irait-il en nu-pied dans cette cohue de grande taille, avec ces billes? 47) Lustrage et pâturage riment un peu plus que fluor et météore. 48) Elle propose des activités de saut kilométrique en altitude à Soumatra. 49) Pis, p, paix, pas, port, peau pou, pu, peux, peur, pan, pont, pain.
S11	50) Très acariâtre. Très acariâtre. Très acariâtre. 51) La bise et le soleil se disputaient chacun assurant qu’il était le plus fort quand ils ont vu un voyageur. 52) Il disputait le voltigeur qui veut de chauds pantalons et des habits de mode sans plis ni go û t. 53) Jouer du biniou électromagnétique ça fait bing contre le givre. 54) Blagues garanties. Blagues garanties. Blagues garanties.
S12	55) Quand la peur se répandit ils ont couru aux voitures enveloppées d’aluminium. 56) Il éblouit le veau et les pioupious qui sautaient à une encablure du Cher. 57) L’humanité uniquement hallucinée, et assoupie par la politique du sous-ministre coula dans l’abîme. 58) Couds ta chemise. Couds ta chemise. Couds ta chemise. 59) Pas de dates précises. Pas de dates précises. Pas de dates précises.
S13	60) Elle a tout faux. Elle a tout faux. Elle a tout faux. 61) Chose inouïe il imita l’anti-roulis sans pâte à choux ni hachis. 62) Les scouts s’enivrent et papillonnent vers les cailloux où le wombat fait la loi sur sa mule. 63) Au milieu du lit où elle dessine des pions sur des cartoonz, le clou rouillé fait un tour. 64) Est-ce un syllogisme de dire que l’homme pédant est un animal mortel.
S14	65) Tout bouffi il dissout la moumoute à l’embouchure de la rivière moussue. 66) Le Chinois républicain Liou cacha les poissons et des agneaux dans la rue. 67) Paule prit les tamtam que la copine utilisera pour annoncer la panne. 68) Puis la structure de l’astragale va glisser doucement dans le ruisseau. 69) Le sextuple adjoint aux sports a un caillot au cerveau.
S15	70) A l'île du saint, la crue du rio vert les submerge tous sans un cri. 71) Nous palissons. Nous palissons. Nous palissons. 72) Infamie suprême, un fou encapuchonné fit mouche avec du gui à la proue. 73) Le truffage du choux nécessite du chiffon et du fil à rouler. 74) Frustré parce-que le cliché est flou, le paranoïaque va là où le climat est meilleur.
S16	75) Avec du culot, la perruquiniste enrichie s’occupa du baby-foot du futur graphiste. 76) Il a pas mal. Il a pas mal. Il a pas mal. 77) Des abat-jour. Des abat-jour. Des abat-jour.

**Online-only Table 2:** List of vowels and silent positions used for 3D static MRI.

Forler name	Phoneme or position	Word-example
UP	Tongue pushed against upper teeth	
DOWN	Tongue pushed against lower teeth	
CONTACT	Incisors in contact	
i	/i/	pis
e	/e/	p (letter of the French alphabet)
E	/ε/	paix
a	/a/	pas
A	/a/ exaggeratedly opened	
O	/ɔ/	port
o	/o/	peau
u	/u/	pou
y	/y/	pu
2	/ø/	peux
9	/oe/	peur
an	/$\widetilde{\alpha }$/	pan
on	/$\widetilde{o}$/	pont
en	/$\widetilde{\varepsilon }$/	pain
retro	Tongue in retroflex position	
deep	Tongue in very retroflex position	

**Online-only Table 3:** List of consonants pronounced in context of different vowels used for 3D static MRI.

Folder name	Phoneme	In context of which phoneme	Word-example
li	/l/	/i/	lit
la	/l/	/a/	la
lu	/l/	/u/	loup
ly	/l/	/y/	lu
len	/l/	/$\widetilde{\varepsilon }$/	plante
ri	/ʁ/	/i/	riz
ra	/ʁ/	/a/	rat
ru	/ʁ/	/u/	roue
ry	/ʁ/	/y/	rue
ren	/ʁ/	/$\widetilde{\varepsilon }$/	rein
pi	/p/	/i/	pis
pa	/p/	/a/	pas
pu	/p/	/u/	pou
py	/p/	/y/	pu
ti	/t/	/i/	titi
tE	/t/	/$\varepsilon$/	tais
ta	/t/	/a/	ta
to	/t/	/o/	tôt
tu	/t/	/u/	tout
ty	/t/	/y/	tu
ki	/k/	/i/	qui
kE	/k/	/ε/	quai
ka	/k/	/a/	cadeau
ko	/k/	/o/	colonie
ku	/k/	/u/	cou
ky	/k/	/y/	cul
k2	/k/	/ø/	queue
kan	/k/	/$\widetilde{a}$/	quand
ken	/k/	/$\widetilde{\varepsilon }$/	quinconce
kon	/k/	/$\widetilde{{\rm{o}}}$/	con
shi	/ʃ/	/i/	Chili
shE	/ʃ/	/ε/	chaise
sha	/ʃ/	/a/	chat
sho	/ʃ/	/o/	chaud
shu	/ʃ/	/u/	choux
shy	/ʃ/	/y/	chuter
sh2	/ʃ/	/ø/	cheveu
si	/s/	/i/	si
sE	/s/	/ε/	sait
sa	/s/	/a/	sa
so	/s/	/o/	sceau
su	/s/	/u/	sous
sy	/s/	/y/	su
s2	/s/	/ø/	ceux
fi	/f/	/i/	fit
fa	/f/	/a/	fa
fu	/f/	/u/	fou
mi	/m/	/i/	mie
ma	/m/	/a/	ma
mu	/m/	/u/	mou
mon	/m/	/$\widetilde{{\rm{o}}}$/	mon
ni	/n/	/i/	ni
na	/n/	/a/	na
nu	/n/	/u/	nous
non	/n/	/$\widetilde{{\rm{o}}}$/	non
ji	/j/	/i/	yiddish
wa	/w/	/a/	voiture

**Online-only Table 4:** List of repetitions or mistakes produced by the speakers during the dynamic 2D MRI.

Patient code	Series	Sentence	Comments
P1	1	1	Series 1. Sentence 1. Repetition of “Le filou et la fripouille”
	2	9	Additional “dans le grand la” before “dans le”
	4	17	Repetition of “l’ine” before “l’incunable”
	8	37	Additional “redoutée par les » before “redoutée par des”
	9	44	Additional “j’” before “j’ai”. Additional “pour voi” before “pour avoir”
	11	53	Additional “Le joureur non” before “Jouer du biniou”
	15	74	Additional “prace” before “parce-que”
P2	1	3	Repetition of “des” before “yaourts”
	4	17	Repetition of “les” before “les outils”
	5	23	“l’humuste” instead of “l’humus”, “dombe” instead of “bombe”
	6	27	Additional “atourdi” before “alourdi »
	7	33	Repetition of “cache”
	8	39	Additional “a” before “sans”, additional “avec l’hu” before “avec humour”
	12	56	Additional “du Fur” before “du Cher”
	13	62	Additional “et le” before “où”
	15	73	Repetition of “à” before “rouler”
P3	1	2	“clamoufla” instead of “camoufla”
	1	4	“lis” instead of “ris”
	4	17	“ouvra l’incurable” instead of “ouvrit l’incunable”, “de Manouches” instead of “des Manouches”
	5	23	“analysons” instead of “analysions”, “cruo” instead of “courroux”, “dit bois” instead of “du bois”
	6	27	“assourdi” instead of “alourdi”, “doit” instead of “dut”
	7	33	“tricotant” instead of “fricotant”
	10	46	“lirait-il” instead of “irait-il »
	11	51	“chacune” instead of “chacun”
	11	53	“Inuer” instead of “jouer”
	13	63	“cartons” instead of “cartoonz”
	13	64	“Elle est” instead of “Ect-ce”
	14	69	“caillou au cerveau” instead of “caillot au cerveau”
	15	74	“lit” instead of “fit”
	16	75	“le perruquiniste” instead of “la perruquiniste”, “a’occupa” instead of “s’occupa”
	16	NA	Additional “Merci” at the end of the series
P4	10	46	“irait t’en” instead of “irait-il en”
	11	52	Repetition of “pa” before “pantalons”
	15	72	Additional “encape” before “encapuchonné”
	16	75	“le perruquiniste” instead of “la perruquiniste”, repetition of “beb” before “baby-foot”
P5	1	4	“ropoussez” instead of “repoussez”
	3	13	Additional “taxs” before “taciturne”
	4	17	Additional “i” before “il ouvrit”
	8	38	“un pou” instead of “du pou”
	11	53	“Joueur” instead of “Jouer”
	12	56	“Il zi” before the sentence
	13	62	“s’en vivrent” instead of “s’enivrent”
	13	64	Repetition of “un syllog” before “un syllogisme”
	16	75	Repetition of “perr” before “perruquiniste”, repetition of “du be” before “du baby-foot”
P6	1	1	Very pronounced “h” at the end of the word “tipi”
	8	39	“azijmut” instead of “azimut”
	10	49	Repetition of “Pis, pé, pas”
	12	59	Repetition of “Pas” during the 2-nd repetition
	13	62	Additional “s” before “loi”
	15	74	“fou” instead of “flou”
P7	3	13	“laciturne” instead of “taciturne”
	4	18	Repetition of “Nous galopâmes”
	6	25	Repetition of “Lui as-tu”
	8	36	Additional “ve” before “vadrouille”
	11	53	“Joueur” instead of “Jouer”
	14	65	“dissut” instead of “dissout”
	14	66	“cache” instead of “cacha”
P8	2	5	Repetition of “l’ocul” before “l’oculiste”, “di dévisa sans scre crupule” instead of “dévisa sans srupule”
	2	8	“bain” instead of “bassin”
	3	12	“chouchen” instead of “chouan”
	3	14	“des plumes” instead of “les plumes”
	4	15	“sans-nutri” instead of “sous-nutri”
	4	19	“l’accusa” instead of “l’accuse”
	5	23	Additional “tu vou” before “tu voyais”, “couscous” instead of “coucous”
	6	25	Additional “des Zouzou” before “des Zouzous”
	6	26	“accrocha” instead of “accoucha”
	6	28	“Vésus” instead of “Vésuve”
	7	31	Absent “et” before “des ficelles”
	7	33	Additional “ses” before “et ses vices”
	8	38	“pu” instead of “pou”
	8	39	Additional “sans hach” before “sans hachurer”
	9	44	“supernatural” instead of “supranatural”
	10	45	Additional “de” before “du port »
	10	46	“ira-t-il” instead of “irait-il”
	12	55	Additional “se repan” before “se répandit”
	12	58	Additional “Couds da” at the beginning of the first repetitions
	14	66	Additional “cracha les poissons eh” before “cacha”
	14	67	“tatam” instead of “tamtam”
	14	69	Multiple repetitions of “caillot au cerveau”
	15	70	“de rio” instead of “du rio”, repetition of “dans un cri”
	15	74	Additional “le patro” before “le paranoïaque”
	16	75	Additional “s’o” before “s’occupa”
P9	1	2	Additional “choux” before “rouge”
	1	4	Additional “de” before “à la pomme”
	2	7	“on” instead of “ou” before “n’a pas”
	6	23	“chahuter” instead of “chuter”
	7	30	“groupsse” instead of “groupe”
	7	34	“juxtaposaient” instead of “juxtaposeraient”
	8	36	“retour” instead of “détour”
	8	37	“le squaw” instead of “la squaw”
	9	40	“répandant” instead of “réparant”
	9	44	“fi” instead of “fil”
	10	46	“pied nu” instead “nu-pied”
	11	51	Absent “le” before “plus fort”
	12	58	“cheminée” instead of “chemise” (all the 3 repetitions)
	13	62	“mute le” instead of “mule”
	14	66	“récpublicain” instead of “républicain”
	15	70	“de” instead of “du” before “saint”, “et le submerge” instead of “les submerge tous”
	15	74	“passe que” instead of “parce-que”
P10	2	2	Series 2. Sentence 2. Additional “le” before “de loi”.
	2	3	“Cucuptané” instead of “cutané”.
	5	21	“Crag Crabes bagarreurs.” instead “Crabes bagarreurs.” during the 3-rd repetition
	5	23	Additional “des cous” before “des coucous”.
	6	26	Additional “dans les jout” before “dans les choux”.
	7	34	Additional “jus” before “juxtaposeraient”
	8	38	Additional “pour lui en” before “pour les enfants”
	10	46	“Irais-tu” instead of “irait-il”
	10	48	Additional “à Sam” before “à Soumatra”
	10	49	Swapped “pu” and “pou”
	11	53	“des bingues” instead of “bing”
	13	62	“wombe” instead of “wombat”
	14	69	Repetition of “au” before “au cerveau”
	15	70	“le submerge” instead of “les submerge”, additional “dans” before “tout sens”.
	15	74	“pasque” instead of “parce-que”
