# Supplementary material for: Multimodal dataset of real-time 2D and static 3D MRI of healthy French speakers
Source: Sci Data. 2021 Oct 1;8:258. doi: 10.1038/s41597-021-01041-3 (PMC8486854; doi:10.1038/s41597-021-01041-3)
Supplement: Supplementary file 1 — Supplementary Material [file 41597_2021_1041_MOESM1_ESM.pdf]

Acquisitions dynamiques

- Les phrases sont données par liste de 5 et ensuite 1 par slide.
- Quelques prononciations à respecter:
  - Paule -- /pol/ o fermé
  - Clown -- /klun/
  - Squaw -- /skwo/
  - Sumatra – /sumatra/ (avec un ou)
  - Cartoons – /kartunz/ (avec un ou)
  - Perruquiniste – /per**y**kinist/
  - Wombat – /wombat/ comme « wombate »
- Il faut ne pas faire de pause et donc enchaîner les mots sauf s'il y a une virgule.

1) Le filou et la fripouille manipulent de l'acrylique antirides dilué sous le tipi.
2) En haut du cumulus Pierre prit dix choux, du rouge et du clafoutis puis se camoufla en clown.
3) L'actionnaire des yaourts Caprice des Dieux couvrit le doigt sur le cahier aimanté.
4) Ne repoussez pas l'écrou de la galtouse de ris à la pomme.
5) Du coup l'oculiste tout fou dévissa sans scrupule le volant du véhicule.

6) La ciguë de l'homme de loi roux est dans la grue sur le parking.
7) Il n' a pas voulu, ou n'a pas pu injecter un sous-multiple de la dose en sous-cutané.
8) Je veux annuler pour éviter le raffut du saut dans le grand bassin.
9) Plus nous y croyons pire est le trou dans le lit du pauvre.
10) Trois sacs carrés. **– à répéter 3 fois-**

11) Vous dactylographiez sa soupe sirupeuse au lit.
12) Le chouan qui parle wolof, et a l'ouïe fine prépare une mixture bien pire.
13) Le chouchou du fou truqua le chargeur du fusil de leur nounou taciturne.
14) Sonne le glas à plat sans faire glouglou dans le foin et les plumes.
15) Le stupide toutou sous-nutri anticipa couci-couça l'africanisation des bikinis.

16) Les attabler.**– à répéter 3 fois-**
17) Il pouffa quand il ouvrit l'incunable qui montrait un prunus et les outils des Manouches.
18) Nous galopâmes avec peine jusqu'au bout sous le soleil.
19) Elle l'accuse de la diffamer en disant qu'elle a couru et s'est amusée avec du ciment et du sable humide.
20) J'exultais car elle joue et fume comme jamais avec les poules.

21) Crabes bagarreurs.**– à répéter 3 fois-**
22) Il a pourri.**– à répéter 3 fois-**
23) Nous analysions avec courroux l'humus du bois touffu, où tu voyais des bombes antichars et des coucous.
24) En écoutant la flûte, le chevreau mangea la robe à froufrous de Maurine.
25) Lui as-tu pris ta presse pour les piles du roi des Zoulous ?

26) Elle culbuta et accoucha huit fois dans les choux de Gilles.
27) Drapé dans son manteau mais pas du tout alourdi par le poids du chat il dut chuter sur la mosaïque.
28) Paul jugeait le Vésuve sans danger depuis le môle.
29) Il sut ça si tôt, qu'il fit tout pour diffuser les coupures à ras bord.
30) Des nuages gris et un cyclone destructeur s'approchent du groupe polaire.

31) Je vois le loubard, le wagon et des ficelles qui chutent dans la rivière.
32) Il l'a daté.**– à répéter 3 fois-**
33) A la cantine, un Druze cache ses frasques et ses vices, en fricotant avec un plouc.
34) Au bilan, les députés juxtaposeraient la sous-poutre.
35) Pour tout casser.**– à répéter 3 fois-**

36) Amoindri par les tirs, le flibustier vadrouille à hue et à dia sans détour.
37) Finalement le loup du roi a vu la squaw redoutée des alouettes de Laval.
38) Elle moulut du pou chilien et du loup pour les enfants affamés du ru.
39) L'azimut chimique partira sans hachurer les sinus acquis avec humour pendant la pénurie.
40) L'aménageur qui est venu cherchait l'anthologie des appareils se réparant seuls.

41) L'ouvrage qui disposait d'une boussole était carbonisé de part en part.
42) Le premier des voyous ment très fort avant de souffler sur le nageur.
43) Il zappe pas mal.**– à répéter 3 fois-**
44) Comme alternative, j'ai agglutiné des tours de fil pour avoir un aimant supranaturel.
45) L'exclusivité fait peur à l'administrateur de biens du port.

46) Où irait-il en nu-pied dans cette cohue de grande taille, avec ces billes ?
47) Lustrage et pâturage riment un peu plus que fluor et météore.
48) Elle propose des activités de saut kilométrique en altitude à Soumatra.
49) Pis, p, paix, pas, port, peau pou, pu, peux, peur, pan, pont, pain.
50) Très acariâtre.**– à répéter 3 fois-**

51) La bise et le soleil se disputaient chacun assurant qu'il était le plus fort quand ils ont vu un voyageur.

52) Il disputait le voltigeur qui veut de chauds pantalons et des habits de mode sans plis ni goût.

53) Jouer du biniou électromagnétique ça fait bing contre le givre.

54) Blagues garanties.**– à répéter 3 fois-**

55) Quand la peur se répandit ils ont couru aux voitures enveloppées d'aluminium.

56) Il éblouit le veau et les pioupious qui sautaient à une encablure du Cher.
57) L'humanité uniquement hallucinée, et assoupie par la politique du sous ministre coula dans l'abîme.
58) Couds ta chemise.**– à répéter 3 fois-**
59) Pas de dates précises.**– à répéter 3 fois-**
60) Elle a tout faux.**– à répéter 3 fois-**

61) Chose inouïe il imita l'anti-roulis sans pâte à choux ni hachis.
62) Les scouts s'enivrent et papillonnent vers les cailloux où le wombat fait la loi sur sa mule.
63) Au milieu du lit où elle dessine des pions sur des cartoonz, le clou rouillé fait un tour.
64) Est ce un syllogisme de dire que l'homme pédant est un animal mortel.
65) Tout bouffi il dissout la moumoute à l'embouchure de la rivière moussue.

66) Le Chinois républicain Liou cacha les poissons et des agneaux dans la rue.
67) Paule prit les tamtam que la copine utilisera pour annoncer la panne.
68) Puis la structure de l'astragale va glisser doucement dans le ruisseau.
69) Le sextuple adjoint aux sports a un caillot au cerveau.
70) A l'île du saint, la crue du rio vert les submerge tous sans un cri.

71) Nous palissons.**– à répéter 3 fois-**
72) Infamie suprême, un fou encapuchonné fit mouche avec du gui à la proue.
73) Le truffage du choux nécessite du chiffon et du fil à rouler.
74) Frustré parce-que le cliché est flou, le paranoïaque va là où le climat est meilleur.
75) Avec du culot, la perruquiniste enrichie s'occupa du baby-foot du futur graphiste.

76) Il a pas mal.**– à répéter 3 fois-**
77) Des abat-jour.**– à répéter 3 fois-**

1) Le filou et la fripouille manipulent de l'acrylique antirides dilué sous le tipi.

2) En haut du cumulus Pierre prit dix choux, du rouge et du clafoutis puis se camoufla en clown.

3) L'actionnaire des yaourts Caprice des Dieux couvrit le doigt sur le cahier aimanté.

4) Ne repoussez pas l'écrou de la galtouse de ris à la pomme.

5) Du coup l'oculiste tout fou dévissa sans scrupule le volant du véhicule.

6) La ciguë de l'homme de loi roux est dans la grue sur le parking.

7) Il n'a pas voulu, ou n'a pas pu injecter un sous-multiple de la dose en sous-cutané.

8) Je veux annuler pour éviter le raffut du saut dans le grand bassin.

9) Plus nous y croyons pire est le trou dans le lit du pauvre.

10) Trois sacs carrés

– à répéter 3 fois-

11) Vous dactylographiez sa soupe sirupeuse au lit.

12) Le chouan qui parle wolof et a l'ouïe fine prépare une mixture bien pire.

13) Le chouchou du fou truqua le chargeur du fusil de leur nounou taciturne.

14) Sonne le glas à plat sans faire glouglou dans le foin et les plumes.

15) Le stupide toutou sous-nutri anticipa couci-couça l'africanisation des bikinis.

16) Les attabler.

– à répéter 3 fois-

17) Il pouffa quand il ouvrit l'incunable qui montrait un prunus et les outils des Manouches.

18) Nous galopâmes avec peine jusqu'au bout sous le soleil.

19) Elle l'accuse de la diffamer en disant qu'elle a couru et s'est amusée avec du ciment et du sable humide.

20) J'exultais car elle joue et fume comme jamais avec les poules.

21) Crabes bagarreurs.

– à répéter 3 fois-

22) Il a pourri.

– à répéter 3 fois-

23) Nous analysions avec courroux l'humus du bois touffu, où tu voyais des bombes antichars et des coucous.

24) En écoutant la flûte, le chevreau mangea la robe à froufrous de Maurine.

25) Lui as-tu pris ta presse pour les piles du roi des Zoulous ?

26) Elle culbuta et accoucha huit fois dans les choux de Gilles.

27) Drapé dans son manteau mais pas du tout alourdi par le poids du chat il dut chuter sur la mosaïque.

28) Paul jugeait le Vésuve sans danger depuis le môle.

29) Il sut ça si tôt, qu'il fit tout pour diffuser les coupures à ras bord.

30) Des nuages gris et un cyclone destructeur s'approchent du groupe polaire.

31) Je vois le loubard, le wagon et des ficelles qui chutent dans la rivière.

32) Il l'a daté.

– à répéter 3 fois-

33) A la cantine, un Druze cache ses frasques et ses vices, en fricotant avec un plouc.

34) Au bilan, les députés juxtaposeraient la sous-poutre.

35) Pour tout casser.

– à répéter 3 fois-

36) Amoindri par les tirs, le flibustier vadrouille à hue et à dia sans détour.

37) Finalement le loup du roi a vu la squaw redoutée des alouettes de Laval.

38) Elle moulut du pou chilien et du loup pour les enfants affamés du ru.

39) L'azimut chimique partira sans hachurer les sinus acquis avec humour pendant la pénurie.

40) L'aménageur qui est venu cherchait l'anthologie des appareils se réparant seuls.

41) L'ouvrage qui disposait d'une boussole était carbonisé de part en part.

42) Le premier des voyous ment très fort avant de souffler sur le nageur.

43) Il zappe pas mal.

– à répéter 3 fois-

44) Comme alternative, j'ai agglutiné des tours de fil pour avoir un aimant supranaturel.

45) L'exclusivité fait peur à l'administrateur de biens du port.

46) Où irait-il en nu-pied dans cette cohue de grande taille, avec ces billes ?

47) Lustrage et pâturage riment un peu plus que fluor et météore.

48) Elle propose des activités de saut kilométrique en altitude à Soumatra.

49) Pis, p, paix, pas, port, peau pou, pu, peux, peur, pan, pont, pain.

50) Très acariâtre.

– à répéter 3 fois-

51) La bise et le soleil se disputaient chacun assurant qu'il était le plus fort quand ils ont vu un voyageur.

52) Il disputait le voltigeur qui veut de chauds pantalons et des habits de mode sans plis ni goût.

53) Jouer du biniou électromagnétique ça fait bing contre le givre.

54) Blagues garanties.

– à répéter 3 fois-

55) Quand la peur se répandit ils ont couru aux voitures enveloppées d'aluminium.

56) Il éblouit le veau et les pioupious qui sautaient à une encablure du Cher.

57) L'humanité uniquement hallucinée, et assoupie par la politique du sous-ministre coula dans l'abîme.

58) Couds ta chemise.

– à répéter 3 fois-

59) Pas de dates précises.

– à répéter 3 fois-

60) Elle a tout faux.

– à répéter 3 fois-

61) Chose inouïe il imita l'anti-roulis sans pâte à choux ni hachis.

62) Les scouts s'enivrent et papillonnent vers les cailloux où le wombat fait la loi sur sa mule.

63) Au milieu du lit où elle dessine des pions sur des cartoonz, le clou rouillé fait un tour.

64) Est-ce un syllogisme de dire que l'homme pédant est un animal mortel.

65) Tout bouffi il dissout la moumoute à l'embouchure de la rivière moussue.

66) Le Chinois républicain Liou cacha les poissons et des agneaux dans la rue.

67) Paule prit les tamtam que la copine utilisera pour annoncer la panne.

68) Puis la structure de l'astragale va glisser doucement dans le ruisseau.

69) Le sextuple adjoint aux sports a un caillot au cerveau.

70) A l'île du saint, la crue du rio vert les submerge tous sans un cri.

71) Nous palissons.

– à répéter 3 fois-

72) Infamie suprême, un fou encapuchonné fit mouche avec du gui à la proue.

73) Le truffage du choux nécessite du chiffon et du fil à rouler.

74) Frustré parce-que le cliché est flou, le paranoïaque va là où le climat est meilleur.

75) Avec du culot, la perruquiniste enrichie s'occupa du baby-foot du futur graphiste.

76) Il a pas mal.

– à répéter 3 fois-

77) Des abat-jour.

– à répéter 3 fois-

C’est fini !!!!

MERCI

Acquisitions statiques

Pour ces acquisitions il faut maintenir les articulateurs fixes dans la position qui correspond au son à articuler.
Pour les voyelles il faut produire la voyelle puis arrêter de produire le son en maintenant la position pendant le temps de l'acquisition.
Pour les consonnes, il faut bloquer les articulateurs dans une position qui permette de produire la suite CV indiquée, par exemple /li/, juste après l'acquisition.

1) Langue poussée contre les dents du haut

2) Langue poussée contre les dents du bas

3) Incisives en contact

4) /i/ en pensant au mot « pis »

5) /e/ en pensant à la lettre « p »

6) /ɛ/ en pensant au mot « paix »

7) /a/ en pensant au mot « pas »

8) /a/ en exagérant un peu l’ouverture

9) /ɔ/ en pensant au mot « port »

10) /o/ en pensant au mot « peau »

11) /u/ en pensant au mot « pou »

12) /y/ en pensant au mot « pu »

13) /ø/ en pensant au mot « peux »

14) /œ/ en pensant au mot « peur »

15) /ã/ en pensant au mot « pan »

16 /õ/ en pensant au mot « pont »

17) /ɛ̃/ en pensant au mot « pain »

18) Langue en position rétroflexe (la pointe dirigée vers le haut et l’arrière)

19) Langue en position très rétroflexe (la pointe dirigée vers le haut et l’arrière)

Consonnes

20) /li/ comme dans le mot « lit »

21) /la/ comme dans le mot « la »

22) /lu/ comme dans le mot « loup »

23) /ly/ comme dans « il a lu »

24) /lɛ̃/ comme dans « lin (la plante) »

25) /ʁi/ comme dans le mot « riz »

26) /ʁa/ comme dans le mot « rat »

27) /ʁu/ comme dans le mot « roue »

28) /ʁy/ comme dans le mot « rue »

29) /ʁɛ̃/ comme dans le mot « rein »

30) /pi/ comme dans le mot « pis »

31) /pa/ comme dans le mot « pas »

32) /pu/ comme dans le mot « pou »

33) /py/ comme dans le mot « pu »

34) /ti/ comme dans le mot « titi »

35) /tɛ/ comme dans le mot « tais »

36) /ta/ comme dans le mot « ta »

37) /to/ comme dans le mot « tôt »

38) /tu/ comme dans le mot « tout »

39) /ty/ comme dans le mot « tu »

40) /ki/ comme dans le mot « qui »

41) /kɛ/ comme dans le mot « quai »

42) /ka/ comme dans le mot « cadeau »

43) /ko/ comme dans le mot « colonie »

44) /ku/ comme dans le mot « cou »

45) /ky/ comme dans le mot « cul »

46) /kø/ comme dans le mot « queue »

47) /kã/ comme dans le mot « quand »

48) /kɛ̃/ comme dans le mot « quinconce »

49) /kõ/ comme dans le mot « con »

50) /ʃi/ comme dans le mot « Chili »

51) /ʃɛ/ comme dans le mot « chaise »

52) /ʃa/ comme dans le mot « chat »

53) /ʃo/ comme dans le mot « chaud »

54) /ʃu/ comme dans le mot « choux »

55) /ʃy/ comme dans le mot « chuter »

56) /ʃø/ comme dans le mot « cheveu »

57) /si/ comme dans le mot « si »

58) /sɛ/ comme dans le mot « sait »

59) /sa/ comme dans le mot « sa »

60) /so/ comme dans le mot « sceau »

61) /su/ comme dans le mot « sous »

62) /sy/ comme dans le mot « su »

63) /sø/ comme dans le mot « ceux »

64) /fi/ comme dans le mot « fit »

65) /fa/ comme dans le mot « fa »

66) /fu/ comme dans le mot « fou »

67) /mi/ comme dans le mot « mie »

68) /ma/ comme dans le mot « ma »

69) /mu/ comme dans le mot « mou »

70) /mõ/ comme dans le mot « mon »

71) /ni/ comme dans le mot « ni »

72) /na/ comme dans le mot « na »

73) /nu/ comme dans le mot « nous »

74) /nõ/ comme dans le mot « non »

75) /ji/ comme dans le mot « yiddish »

76) /wa/ comme dans le mot « voiture »

Acquisitions statiques - compléments

77) /le/ comme dans le mot « Léman »

78) /lo/ comme dans le mot « l’eau »

79) /ʁɛ/ comme dans le mot « raie »

80) /ʁo/ comme dans le mot « rot »

81) /ʁɑ̃/ comme dans le mot « rend »

82) /ʁɔ̃/ comme dans le mot « rond »

83) /pɛ/ comme dans le mot « paix »

84) /po/ comme dans le mot « peau »

85) /fɛ/ comme dans le mot « fais »

86) /fo/ comme dans le mot « faux »

87) /fy/ comme dans le mot « fut »

88) /fø/ comme dans le mot « feu »

89) /mɛ/ comme dans le mot « mais »

90) /mo/ comme dans le mot « mot »

91) /my/ comme dans le mot « mue »

92) /nɛ/ comme dans le mot « nais »

93) /no/ comme dans le mot « nos »

94) /ny/ comme dans le mot « nu »
